# *rustybam*: a composable toolkit for alignment analysis and visualization with *SafFire*

**DOI:** 10.1093/bioinformatics/btag799

**Published:** 2026-10-28

**Authors:** Cade Mirchandani, Mitchell R Vollger

**Affiliations:** Department of Human Genetics and Utah Center for Genetic Discovery, University of Utah, Salt Lake City, UT 84112, United States; Department of Human Genetics and Utah Center for Genetic Discovery, University of Utah, Salt Lake City, UT 84112, United States

## Abstract

**Summary:**

We present *rustybam*, a Rust-based command-line toolkit for CIGAR-aware manipulation of PAF and BAM alignments, and *SafFire*, a browser-based tool for interactive genome comparison visualization with annotation overlays. *rustybam’s* composable subcommands provide overlap resolution, coordinate liftover, alignment splitting at structural variants, scaffolding, and identity statistics.

**Availability and implementation:**

Both tools are open source under the MIT license. *rustybam* is available via Bioconda (https://anaconda.org/bioconda/rustybam), crates.io, and GitHub (https://github.com/vollgerlab/rustybam). *SafFire* is hosted at https://vollgerlab.com/SafFire/ with source code at https://github.com/vollgerlab/SafFire. Archived versions are available on Zenodo: rustybam (https://doi.org/10.5281/zenodo.4654875) and SafFire (https://doi.org/10.5281/zenodo.5765964).

## 1 Introduction

Telomere-to-telomere (T2T) assemblies of diploid human genomes and pangenomes have made whole-genome pairwise alignment a routine step in comparative genomics (Nurk *et al.* 2022, Liao *et al.* 2023). The minimap2 aligner (Li 2018) established the Pairwise mApping Format (PAF), now a de facto standard for representing pairwise alignments. However, whole-genome alignment presents challenges at copy-number and structurally variable loci where supplementary alignments are required. In these regions, aligners (including minimap2) can produce overlapping alignments in which the same query bases are redundantly mapped to multiple target positions across different supplementary alignments. This is distinct from secondary alignments, in which the same query sequence is intentionally allowed to align to multiple loci. Without resolution, these overlaps inflate estimates of copy-number variation, confound breakpoint identification, produce misleading visualizations, and confound liftover operations.

A rich ecosystem of tools supports alignment post-processing. paftools.js provides alignment, simple variant calling, statistics, and liftover operations, and wgatools (Wei *et al.* 2025) offers format conversion, visualization, filtering, and more. Additional options are available for visualization including D-GENIES (Cabanettes and Klopp 2018), mummerplot (Marçais *et al.* 2018), ModDotPlot (Sweeten *et al.* 2024), StainedGlass (Vollger *et al.* 2022b), plotsr (Goel and Schneeberger 2022), and SVbyEye (Porubsky *et al.* 2025).

We present *rustybam*, a Rust-based toolkit of composable subcommands for CIGAR-aware manipulation of PAF and BAM alignments, and *SafFire*, a browser-based visualization tool for interactive genome comparisons with annotation overlays. *Rustybam’s* subcommands chain through Unix pipes, allowing users to build tailored workflows. While *rustybam’s* functionality overlaps with many existing toolkits, it provides two unique features. First, unlike coordinate-only tools such as *paftools.js* and *UCSC liftOver, rustybam liftover* walks CIGAR strings to emit a trimmed alignment record spanning exactly the lifted interval, so lifted regions can be piped directly into downstream analyses that operate on alignments. Second, *rustybam trim-paf* uses dynamic programming to prevent query sequences from aligning to more than one position in the target genome, refining breakpoints and estimates of copy-number variation. *SafFire* renders the resulting alignments as interactive miropeats-style (Parsons 1995) ribbon plots with annotation overlays and URL-based view sharing. Both tools have been used extensively in T2T Consortium (Nurk *et al.* 2022, Vollger *et al.* 2022a, Rautiainen *et al.* 2023, Rhie *et al.* 2023) and Human Pangenome Reference Consortium (Liao *et al.* 2023, Vollger *et al.* 2023) publications, e.g. to construct 1:1 syntenic segmental-duplication alignments for genome-wide divergence analysis and to generate reference liftover chains between T2T-CHM13v2.0 and GRCh38.

## 2 Implementation

### 2.1 rustybam

*rustybam* is written in Rust and distributed via Bioconda, crates.io, and pre-compiled binaries. Its core design principle is that all coordinate operations preserve CIGAR string integrity: when an alignment is trimmed, split, or lifted over, the CIGAR string is updated to reflect the exact change, ensuring that downstream identity calculations and variant calls remain accurate. rustybam accepts both cg (CIGAR) and cs (difference string) tags, preserving individual base substitutions and indel sequences that cannot be reconstructed from CIGAR alone.

The toolkit provides subcommands invoked as *rb < subcommand>* (or equivalently *rustybam < subcommand>*), designed to chain through Unix pipes. The key subcommands include:

***liftover*** projects target-coordinate BED intervals onto query coordinates (or vice versa with - -*qbed*) through PAF alignments. Unlike other liftover tools (paftools.js, UCSC liftOver, etc.), which return lifted coordinates only, *rb liftover* outputs a trimmed PAF record with an updated CIGAR string spanning exactly the lifted region. This design enables direct composition with other *rustybam* subcommands: e.g. piping *rb liftover* into *rb stats* computes per-region alignment identity in a single command, allowing users to assess sequence conservation across lifted intervals without additional tools.

***trim-paf*** resolves overlapping query alignments that arise at duplications and inversion boundaries. The algorithm loads all PAF records, identifies query-coordinate overlaps, and uses cumulative-score maximization with configurable match, mismatch, and indel scores to find the optimal split point within each overlapping CIGAR string. The process iterates from the largest overlap until no query base is aligned more than once, producing clean, non-overlapping alignments suitable for breakpoint analysis and visualization (Fig. 1). Because the trimming operates directly on the CIGAR string, the resulting records retain accurate alignment coordinates and identity statistics.

***break-paf*** splits alignment records at insertions or deletions exceeding a user-defined threshold (e.g. 5000 bp). This transforms large alignments into fine-grained alignment segments for downstream visualization.

***orient*** reorients query contigs so that the majority of aligned bases are in the forward orientation, simplifying visualization and downstream analysis. An optional scaffold mode merges multiple query contigs per target into a single pseudo-scaffold ordered by target position.

***stats*** computes per-alignment identity and coverage statistics directly from CIGAR strings into BED format with identity annotations, providing the input format used by SafFire.

**Figure 1:**
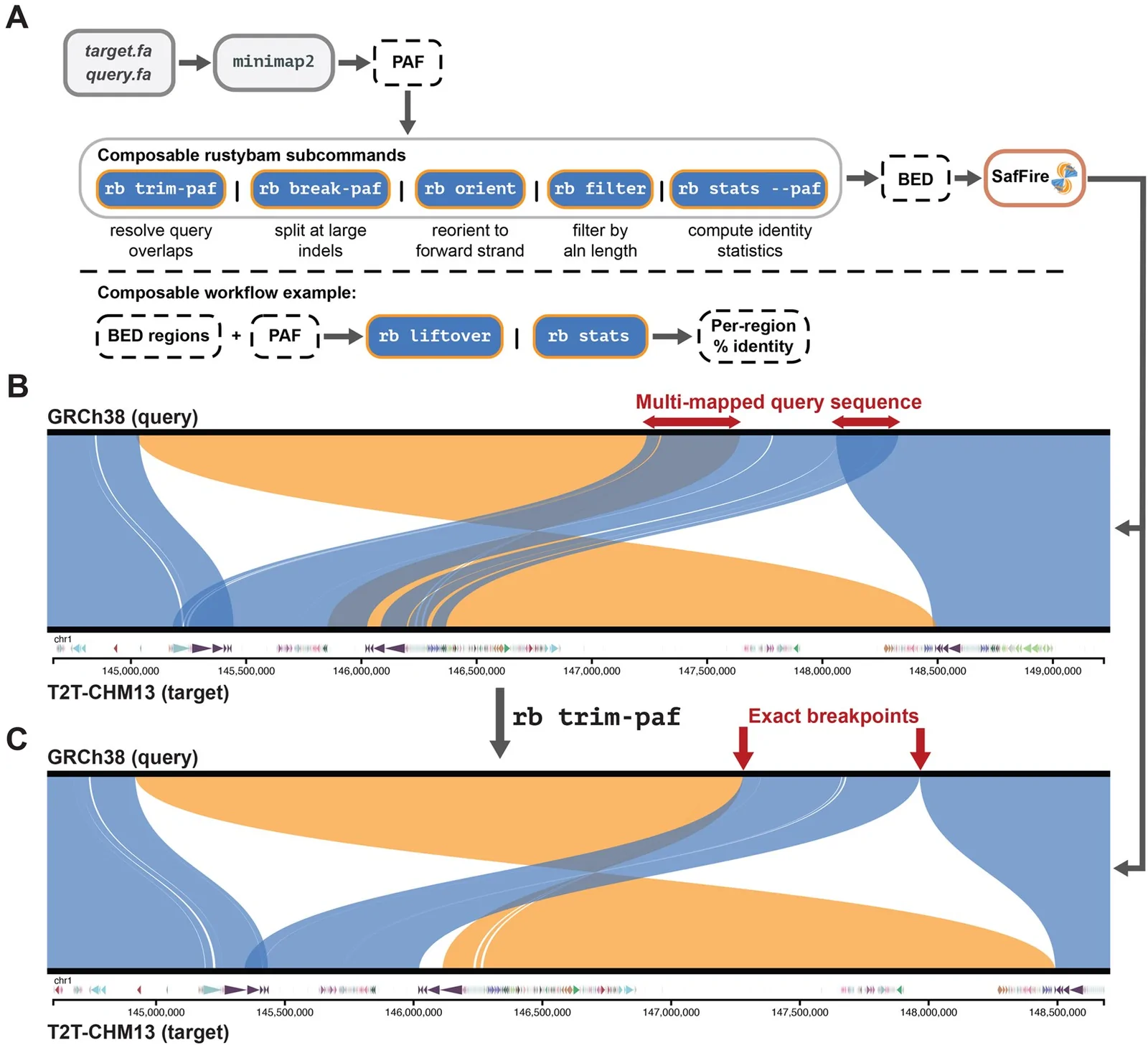
The *rustybam*–*SafFire* pipeline. (A) Schematic of the data processing pipeline from minimap2 PAF through *rustybam* subcommands to *SafFire* visualization. (B) *SafFire* visualization of the *NOTCH2NL* locus on chromosome 1, comparing T2T-CHM13v2.0 (target) and GRCh38 (query). Blue ribbons indicate forward alignments; orange ribbons indicate inversions. BED annotations show segmental duplications annotated by DupMasker. (C) The same locus after applying *rb* t*rim-paf*, showing resolution of overlapping alignments at duplication boundaries visible in (B).

An additional 11 subcommands provide utility operations including PAF filtering, coordinate inversion, sequence extraction, nucleotide frequency calculation, format conversion, and assembly statistics.

A key design goal is that subcommands compose through Unix pipes to build analysis workflows from simple operations. Because subcommands read and write PAF (or BED) through standard streams, users can construct pipelines tailored to specific analyses. For example, lifting gene annotations through an alignment and computing per-gene identity requires only:

rb liftover - -bed genes.bed aln.paf | rb stats - -paf

Similarly, you can combine liftover with overlapping windows to create sliding measures of percent identity, e.g.:

rb liftover - -bed < (bedtools makewindows -g ref.chrom.sizes -w 10000 -s 1000) aln.paf | rb stats - -paf

### 2.2 SafFire

*SafFire* is a browser-based visualization tool that renders miropeats-style (Parsons 1995) genome comparison views as an interactive graphic. It is implemented entirely in client-side JavaScript using D3.js (Bostock *et al.* 2011), requiring no software installation. *SafFire* complements tools like SVbyEye (Porubsky *et al.* 2025) and plotsr (Goel and Schneeberger 2022), which provide miropeats-style plots within R/python workflows, by offering a browser experience with interactivity and URL-based view sharing.

*SafFire* takes as input the BED-format output of rb stats—paf, typically after processing through the *rustybam* pipeline:

minimap2 - -eqx -c -x asm5 ref.fa query.fa\

 | rb trim-paf | rb break-paf -m 5000\

 | rb orient | rb filter - -paired-len 100000\

 | rb stats - -paf > input.bed

Each alignment is rendered as a colored ribbon connecting target and query coordinates: blue for forward alignments, orange for reverse (inversions). Ribbon opacity encodes percent identity, and a percent-identity track below the target axis provides an alignment quality profile. Users can zoom and pan along the target axis, select individual target and query contigs, and click positions to copy genomic coordinates.

Key features include: (i) BED annotation overlays for both target and query sequences, enabling display of gene annotations, centromere satellite classifications, segmental duplication colors, or any custom annotation; (ii) UCSC Genome Browser integration that synchronizes a browser snapshot to the current viewport; (iii) SVG export for publication-quality figures; and (iv) URL-based state sharing via hash parameters (\#ref=&query=&pos=), allowing specific views to be bookmarked and shared. *SafFire* includes pre-loaded datasets with alignments to CHM13 and GRCh38 references, including HPRC and non-human primate assemblies.

## 3 Results

### 3.1 Performance

To evaluate *rustybam’s* performance (v0.2), we benchmarked key subcommands on whole-genome PAF alignments generated by minimap2 (- -eqx -c -x asm5) between CHM13v2 and GRCh38 (1460 records, 69 MB). For less intensive subcommands, we used a 20×-concatenated version of this PAF (29 200 records, 1372 MB) to ensure wall times exceeded measurement noise. All benchmarks used hyperfine with 2 warmup and 5 timed replicates on a MacBook Pro (Apple M4, 64 GB RAM) running single-threaded (-t 1).

*trim-paf* resolved all overlapping alignments across the whole genome in 8.9 s (±0.1 s) on the 1× input. On the 20× input, break-paf completed in 8.6 s (±0.4 s) at a 1000 bp threshold and 10.5 s (±1.1 s) at 5000 bp, orient in 8.6 s (±0.5 s), and filter in 8.6 s (±0.5 s). For alignment statistics, *rb stats* and paftools.js stat showed comparable performance: *rb stats* was slightly faster on the 1× input (155 ms versus 207 ms for paftools.js) while paftools.js was slightly faster on the 20× input (2.7 s versus 3.4 s for rb stats), likely due to their different parsing approaches.

### 3.2 Liftover accuracy

We compared *rb liftover* against paftools.js liftover on 14 565 BED regions lifted through an alignment of T2T-CHM13 to GRCh38 (using paftools.js -l 0 -q 0). Both tools successfully lifted the same 14 274 regions. For the 14 733 coordinate pairs output by paftools.js, 14 666 (99.5%) had identical coordinates, and the remaining 67 (0.5%) differed by exactly 1 bp at the end coordinate, where paftools.js places the end coordinate 1 bp into an insertion. paftools.js was faster than *rustybam* liftover due to their different output strategies: paftools.js performs a block-based coordinate lookup and returns lifted coordinates, while rb liftover walks and trims the full CIGAR string to produce a truncated alignment record that can be piped directly into downstream PAF analysis such as *rb stats*.

### 3.3 Biological application and genome-wide overlap resolution

Figure 1 shows the *rustybam*–*SafFire* pipeline applied to the *NOTCH2NL* locus on chromosome 1, a medically important region containing segmental duplications that differ between GRCh38 and CHM13 (Mefford *et al.* 2008, Vollger *et al.* 2022a). The *trim-paf* step resolves overlapping alignments at duplication boundaries, preventing double-counting in copy-number estimates and enabling detection of base-pair-precise breakpoints. The *SafFire* visualization reveals the complex pattern of repeated and inverted duplications, with BED overlays highlighting segmental duplication classifications.

To quantify the genome-wide impact of overlap resolution via *rb trim-paf*, we aligned the paternal haplotype (query) of HG002 to its maternal haplotype (reference) from the Q100 v1.1 assembly (Hansen *et al.* 2025) and applied *rb trim-paf* (Fig. 1, available as supplementary data at *Bioinformatics* online). In the untrimmed alignment, 31.5 Mbp of the query sequence aligns to more than one reference position, resulting in ambiguous liftover coordinates and inflated estimates of copy-number variation. For example, before trimming, 41.4 Mbp of the reference is covered by two or more query alignments, indicating copy-number gains in the query haplotype; after trimming, this becomes 28.9 Mbp, a 12.5 Mbp reduction in spurious copy-number signal (Fig. 2, available as supplementary data at *Bioinformatics* online). Because these overlaps distort copy-number and liftover estimates genome-wide, as quantified above, resolving them with *trim-paf* is a prerequisite for many downstream analyses.

## 4 Conclusion

*rustybam* and *SafFire* extend the comparative genomics toolkit with CIGAR-aware alignment manipulation and interactive visualization. *rustybam’s* core contributions—overlap resolution (*trim-paf*) and alignment-preserving liftover (*liftover*) complement existing tools such as paftools.js and wgatools. *SafFire* adds interactivity with annotation overlays and URL-based view sharing alongside visualization tools like D-GENIES, SVbyEye, and ModDotPlot, and plotsr. Both tools have been used in T2T Consortium and HPRC publications (Nurk *et al.* 2022, Vollger *et al.* 2022a, 2023, Liao *et al.* 2023, Rautiainen *et al.* 2023, Rhie *et al.* 2023), accumulating over 80 000 downloads on Bioconda and crates.io.

## Supplementary Material

btag799_Supplementary_Data

## Data Availability

All software is open source (MIT license). *rustybam* is archived on Zenodo (DOI: 10.5281/zenodo.4654875) and *SafFire* on Zenodo (DOI: 10.5281/zenodo.5,765,964). Example datasets and a hosted *SafFire* instance are available at https://vollgerlab.com/SafFire/.
