# Supplementary material for: rustybam: a composable toolkit for alignment analysis and visualization with SafFire
Source: Bioinformatics. 2026 Oct 28;42(11):btag799. doi: 10.1093/bioinformatics/btag799 (PMC13739056; doi:10.1093/bioinformatics/btag799)
Supplement: Supplementary file 1 [file btag799_supplementary_data.pdf]

Supplementary Material: *rustybam*: a composable toolkit for alignment analysis and visualization with *SafFire*

Cade Mirchandani[1], Mitchell R. Vollger[1,*]

[1] Department of Human Genetics and Utah Center for Genetic Discovery, University of Utah, Salt Lake City, UT, USA

[*] Corresponding author: mrvollger@genetics.utah.edu

Whole-genome alignment and trimming of the maternal and paternal haplotypes of HG002.

To demonstrate the impact of *trim-paf* genome-wide, we aligned the paternal haplotype (query) of HG002 to the maternal haplotype (reference) of the HG002 Q100 v1.1 assembly with *minimap2* (v2.31-r1302; -x asm5 --eqx -c) and then resolved overlapping query alignments with *rb trim-paf -r* using default scoring parameters. Aligning the two haplotypes of a diploid genome is among the most common whole-genome alignment tasks, and the HG002 Q100 v1.1 assembly provides two near-complete, highly accurate haplotypes in which multiply-aligned query bases reflect overlapping alignments at repeats, duplications, and inversions rather than assembly artifacts.

We quantified the effect of trimming on both the query and the reference haplotypes. For each, we computed per-base coverage over the alignment intervals with *bedtools genomecov* (v2.31.1; -bg) and summed the bases at coverage depth ≥ 2. On the query, these are bases aligning to more than one reference position. The untrimmed alignment contained 31.5 Mbp of such redundancy, which produces ambiguous liftover coordinates; *rb trim-paf* reduces this ambiguity to zero, leaving every query base mapped to a single reference position. On the reference, bases at depth ≥ 2 represent apparent copy-number gain in the query haplotype, which fell from 41.4 Mbp before trimming to 28.9 Mbp after, a 12.5 Mbp reduction in spurious copy-number signal. We show a representative locus before and after trimming in Fig. S1, and a genome-wide summary in Fig. S2.

SafFire input scale and performance.

SafFire renders alignments as SVG using D3.js, so interactive performance depends on the number of alignment segments drawn rather than on raw input file size. The segment count is controlled primarily by *rb break-paf*, which splits alignments at indels larger than a given threshold. We recommend a default threshold of 5,000 bp for genome-wide views and caution against values below the standard structural-variant size of 50 bp, as smaller thresholds break alignments on minor indels and generate many short segments that increase the rendered element count without adding meaningful detail. With this setting, the standard rustybam preprocessing pipeline, and selection of individual target and query contigs for display, whole-genome datasets remain responsive in a standard browser.

Supplementary Figures

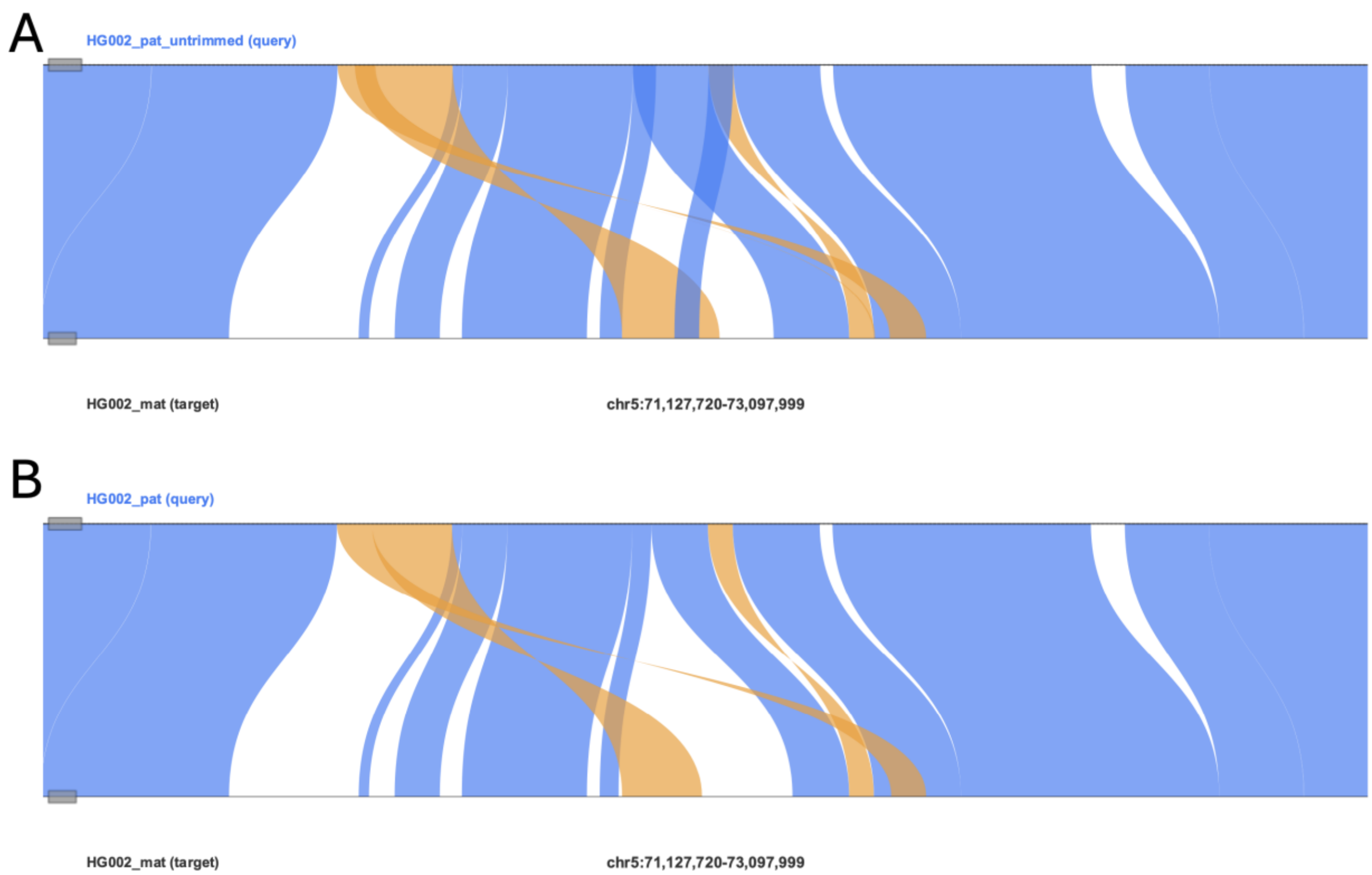

Supplementary Figure S1. SafFire visualization of the HG002 Q100 v1.1 maternal–paternal alignment at chr5:71,127,720-73,097,999. (A) Before and (B) after *rb trim-paf*, showing resolution of the overlapping alignments at duplication and inversion boundaries.

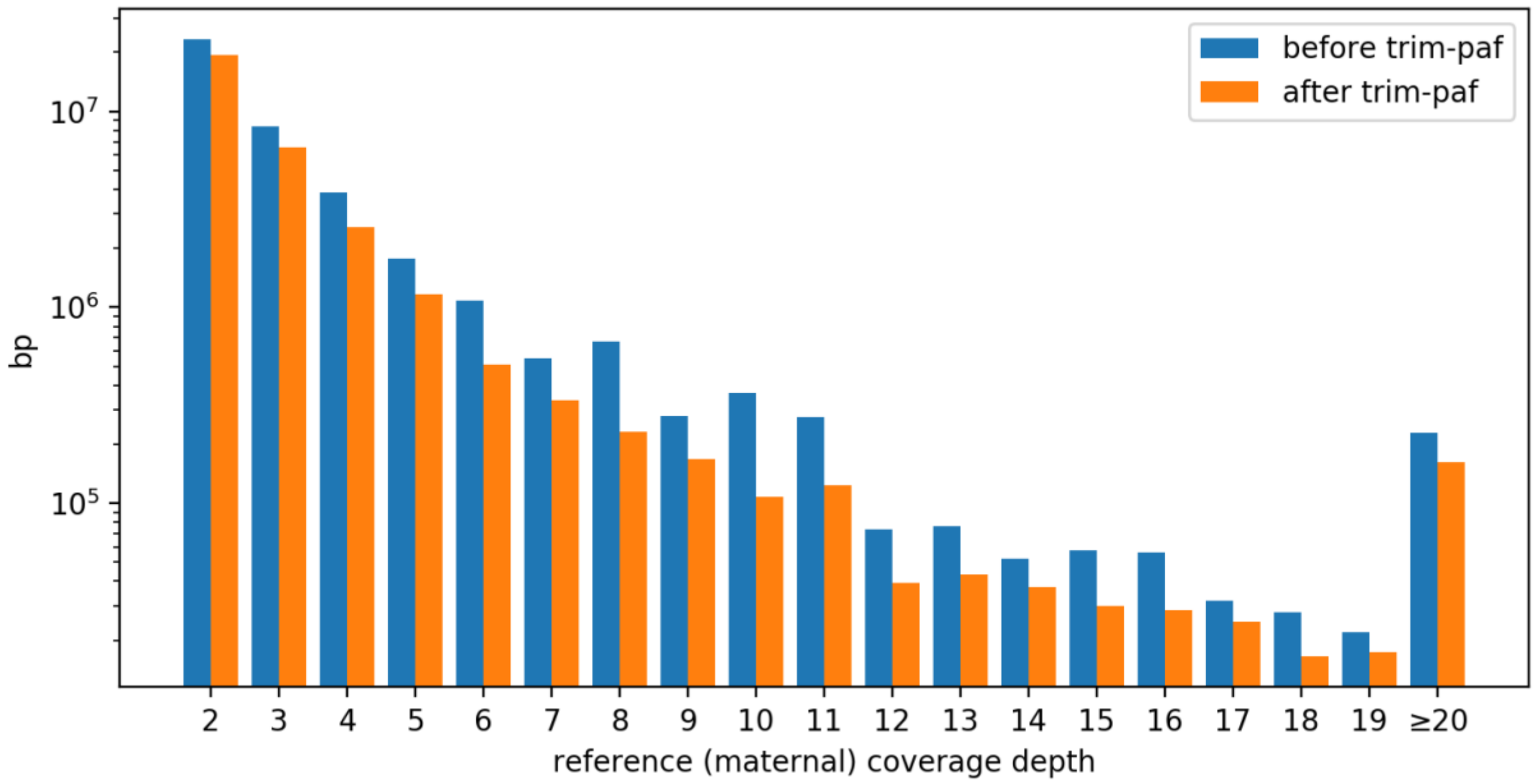

Supplementary Figure S2. Reference coverage depth in the HG002 Q100 v1.1 maternal–paternal alignment, before and after rb trim-paf.
